# The Remission Effects of First Injection of Sclerotherapy for Pediatric Rectal Prolapse: A Systematic Review and Meta-Analysis

**DOI:** 10.3389/fsurg.2022.835235

**Published:** 2022-02-24

**Authors:** Weimo Zhou, Yingzuo Shi, Ming Zhang, Li Li

**Affiliations:** ^1^Department of Pediatric Surgery, Guigang City People's Hospital, Eighth Affiliated Hospital of Guangxi Medical University, Guigang, China; ^2^Department of Pediatric Surgery, Wuxi People's Hospital, Wuxi, China; ^3^Clinical Laboratory, Zhucheng Maternal and Child Health Hospital, Zhucheng, China; ^4^The Department of Pediatrics, Ganzhou People's Hospital, Ganzhou, China

**Keywords:** pediatric, rectal prolapse, sclerotherapy, first injection, remission, meta-analysis

## Abstract

**Background:**

Pediatric rectal prolapse is a common issue in clinical practice. Among various managements, sclerotherapy is an important method to successfully treat pediatric rectal prolapse, especially for the first injection. The knowledge of the first injection of sclerotherapy can be revealed by a systemic review and meta-analysis of randomized clinical trials.

**Methods:**

We performed a systematic search and a meta-analysis for the retrospective clinical studies of sclerotherapy in pediatric rectal prolapse. The comparison between remission and recurrence after the first injection of sclerotherapy was performed to find if the first injection of sclerotherapy can treat rectal prolapse completely. After a restricted selection, 17 studies involving 1,091 pediatric rectal prolapse subjects with sclerotherapy were enrolled in a variety of classifications of injection agents. The focused outcome was to check whether the first injection of sclerotherapy can achieve a remission status. The meta-analysis was performed by Review Manager 5.4.

**Results:**

Among the subjects receiving sclerotherapy, the meta-analysis favors the remission status after receiving the first injection of sclerotherapy. The meta-analysis results showed significant remission tests for the overall effect and significant heterogeneities in odds ratio and the fixed-effects model. The significant therapeutic effects remained, however, even after testing in the relative risk and the random-effects model.

**Conclusions:**

Despite significant heterogeneity and relatively low quality of evidence, the first injection of sclerotherapy may conceivably demonstrate therapeutic effects to help the patients of pediatric rectal prolapse achieve a remission status.

## Introduction

Pediatric rectal prolapse is a significant issue in clinical practice. The weak pelvic musculature might predispose the children to such disease. In addition, the loose attachment of rectal mucosa to the muscularis will contribute to rectal prolapse (1). Most patients of pediatric rectal prolapse belong to the mucosal subtype, which is related to straining due to constipation during the toilet training process in children. Pediatric rectal prolapse in older children might be related to congenital neuromuscular abnormalities, autism or developmental delay, and anorectal malformations (2, 3).

The incidence rate of pediatric rectal prolapse was still not clear (4, 5). Spontaneous resolution would be around 60–90% in pediatric rectal prolapse. Compared to pediatric rectal prolapse, rectal prolapse in adults was rarely spontaneously resolving (2). Despite the high rate of spontaneous resolution, pediatric patients with rectal prolapse will suffer much if the condition will not spontaneously resolve. Therefore it is still necessary to receive the management if there is no spontaneous resolution. Before the ultimate choice of operation (6, 7), sclerotherapy would be an important procedure to relieve the pediatric prolapse (3, 5, 8–11). Therefore, it is important for us to understand the intermediate choice (sclerotherapy) between operation and conservative treatment. In this study, we planned to enroll the related articles of sclerotherapy in pediatric rectal prolapse. We wanted to confirm the remission effects of the first injection of sclerotherapy in pediatric rectal prolapse in the study design of systematic review and meta-analysis. We hypothesized that the first injection of sclerotherapy would favor remission rather than recurrence (no remission after the first injection of sclerotherapy and necessary subsequent injection of sclerotherapy).

## Methods

### Literature Search and Selection Criteria

We used the following keywords: “rectal” or “rectum” or “prolapse” or “children” or “pediatric” or “sclerotherapy” and “rectal prolapse” to search and to collect the related articles in the PubMed, ScienceDirect, Embase, Web of Science, and Scopus databases. The articles were limited to those published or e-published online before December 2021.

The inclusion criteria of this study were as follows: (1) Sclerotherapy treatment for pediatric patients with rectal prolapse; (2) The studies with sclerotherapy outcome and related clinical profiles; (3) The studies with detailed data of sclerotherapy and pediatric rectal prolapse; (4) These studies were also published in English language in the journals of science citation index database; and (5) Retrospective or prospective study. The exclusion criteria were the following: (1) Some parts of detailed data were unavailable in the content of the articles (unavailable even after inquiring the corresponding authors about the data needed for this meta-analysis); (2) The authors did not respond or could not have access to the dataset, in which case the articles would be excluded as the category without detailed data; (3) The studies that do not belong to patients with rectal prolapse or sclerotherapy; and (4) Review articles.

### Quality Assessment and Data Extraction

The study was conducted according to the preferred reporting items for systematic reviews and meta-analyses (PRISMA) guidelines (23). The risk of bias for each study was assessed by the random sequence generation, allocation concealment, blinding of participants and personnel, blinding of outcome assessment, incomplete outcome data, and selective reporting. We extracted the following data from the eligible articles. First, the success rate and patient number of the first injection of sclerotherapy in pediatric patients with rectal prolapse were gathered. Second, the remission rate and patient number under the first injection of sclerotherapy were collected. Third, the recurrence and patient number of the first injection of sclerotherapy in pediatric patients with rectal prolapse were gathered. Fourth, the probable side effects of sclerotherapy in pediatric patients with rectal prolapse was noted.

### Data Extraction and Critical Appraisal

WZ reviewed the abstracts to screen the articles. WZ and YS performed the extraction of clinical outcome data from the text, tables, and figures of the enrolled articles independently. The enrolled articles had the clinical outcome data in the text, tables, figures, or supplementary material. Then, a collaborative review was performed to resolve any discrepancies. All authors participated to review the final results.

### Meta-Analysis and Statistical Analysis

The odds ratio (OR) with 95% CI (a summary statistic) was obtained by the Mantel–Haenszel method for dichotomous variables. Chi-square tests were used to study heterogeneity between the enrolled studies. The derived I^2^ statistic was used to estimate the statistical heterogeneity of studies included in the meta-analysis. The I^2^ statistic was used to estimate the percentage of the total variation across studies due to heterogeneity rather than chance. The I^2^ values of 25, 50, and 75% represented low, medium, and high heterogeneity, respectively. If the heterogeneity is high, the random-effects model will be used for the analysis and the fixed-effects model will be used for studies with low or moderate heterogeneity. Subgroup analyses were not possible due to the lack of patient-level data. All *P* values were two-sided. All statistical analyses were conducted with Review Manager Version 5.4 (The Cochrane Collaboration, London).

## Results

### Description of Studies

**Figure 1:**
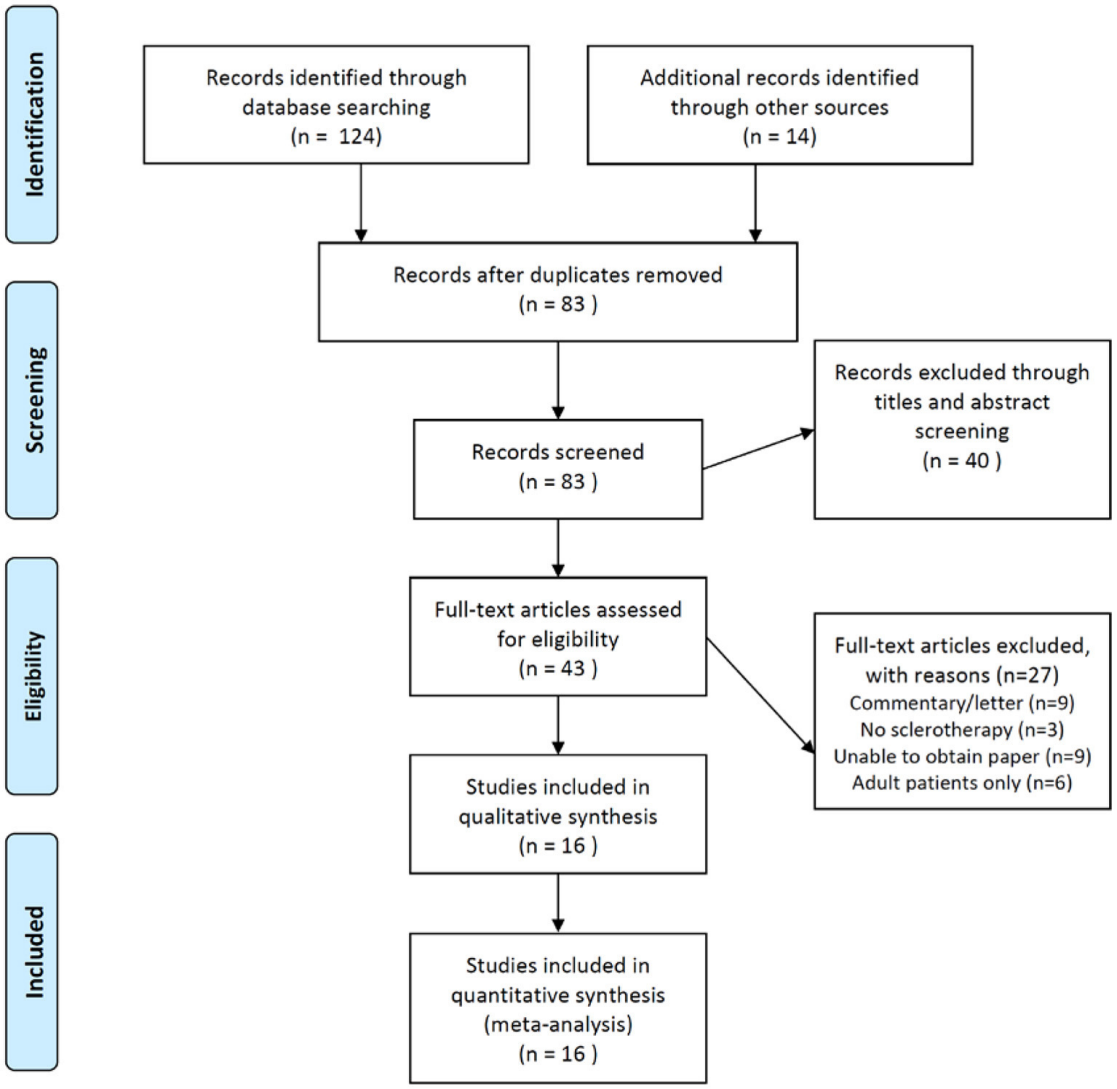
The preferred reporting items for systematic reviews and meta-analyses (PRISMA) flow diagram of the current meta-analysis. The current meta-analysis followed the PRISMA guideline to identify the potentially relevant literature and to screen the identified literature using the abstract and title selection. The full text of screened literature was assessed to find the eligible studies and to include the suitable ones for the final meta-analysis.

The initial literature search through the dataset found 124 articles and the additional records from other sources were 14 articles. Then, 55 duplicates were removed and the residual 83 articles were screened according to the relevance of abstracts and titles. Of that, 40 articles were discarded after this step. Full-text contents of the remaining 43 articles were assessed for eligibility. Then, 27 articles were excluded due to review articles, not nurse-led studies, not randomized trials, and not perioperative setting. The qualitative analysis of these 16 articles was performed and no articles were excluded. Therefore, only 16 studies were included in this meta-analysis (1, 4, 5, 10–22). The flow diagram was presented according to the PRISMA guideline (Figure 1). The detailed characteristics of the 16 studies were also summarized in Table 1.

**Table 1:** Summary of enrolled studies for sclerotherapy of pediatric rectal prolapse.

	**Study design**	**Subjects**	**Injection agent**	**Remission vs. recurrence**
Abes and Sarihan (12)	Retrospective	Rectal prolapse	Normal saline	15 vs. 1
Antao et al. (13)	Retrospective	Rectal prolapse (median age: 3.3 years (4 months−10 years)	Phenol	7 vs. 17
Bahador et al. (14)	Retrospective	Rectal prolapse (108 male, 45 female) (mean age: 2.2 years (9 months to 5 years).	96% ethyl alcohol	147 vs. 6
Batool et al. (15)	Retrospective	Prolonged rectal prolapse	Phenol in almond oil	29 vs. 21
Chan et al. (5)	Retrospective	Rectal prolapse	D50 water	9 vs. 5
Dolejs et al. (4)	Retrospective	Rectal prolapse	Phenol in peanut oil	35 vs. 16
Fahmy and Ezzelarab (16)	Retrospective	Rectal prolapse	Ethyl alcohol, phenol in almond oil, deflux	78 vs. 7
Freeman (1)	Retrospective	Rectal prolapse	Phenol in almond oil	18 vs. 0
Kay and Zachary (11)	Retrospective	Rectal prolapse (29 males, 22 females)	Normal saline	40 vs. 11
Malyshev and Gulin (17)	Retrospective	Rectal prolapse	Ethyl alcohol	339 vs. 14
Sahay et al. (18)	Retrospective	Rectal prolapse	Phenol	16 vs. 7
Sarmast et al. (19)	Retrospective	Rectal prolapse	Normal saline	48 vs. 2
Sasaki et al. (20)	Restrospective	Rectal prolapse 5 boys, 4 girls mean age: 6.5 years; (2.5–14 years)	Phenol in almond oil	6 vs. 3
Shah et al. (21)	Retrospective	Rectal prolapse median age: 2.5 years; (2–4.5 years)	Normal saline	12 vs. 5
Wyllie (10)	Retrospective	Rectal prolapse average age: 2.5 years	Phenol in almond oil	86 vs. 5
Zganjer et al. (22)	Retrospective	Rectal prolapse mean age: 6.2 years; (4–8 years)	Cow's milk	80 vs. 6

### Odds of Remission for the First Injection of Sclerotherapy in Pediatric Rectal Prolapse

**Figure 2:**
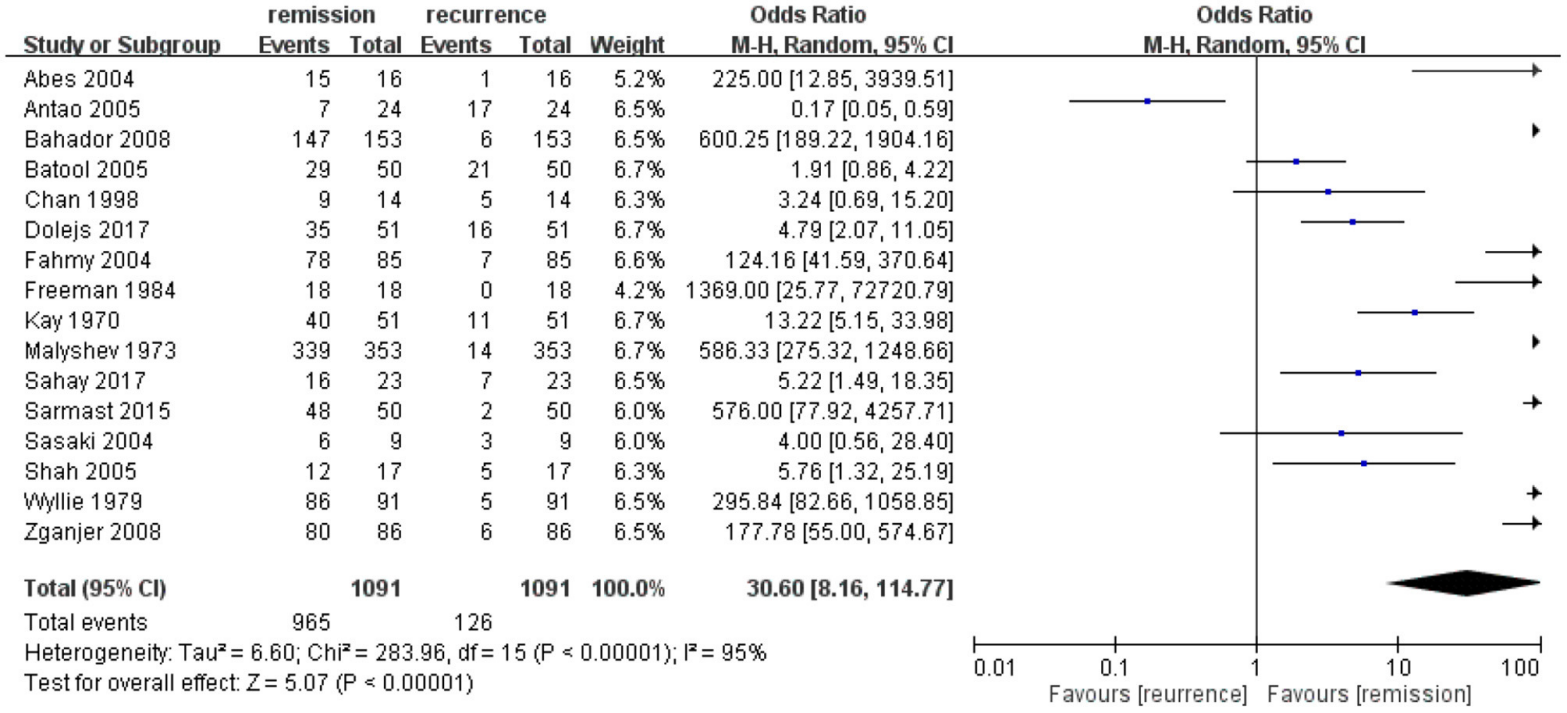
The odds ratio of remission vs. recurrence for the first injection of sclerotherapy in the enrolled studies of pediatric rectal prolapse. The first injection of sclerotherapy showed a favorable result toward remission rather than recurrence. The heterogeneity was high and the result was statistically significant.

The I^2^ was 95% CI, which revealed high heterogeneity. Therefore, the random-effects model was applied. The test for the overall effect was Z = 5.07 (*p* < 0.00001) and the meta-analysis results favored remission after the first injection of sclerotherapy in pediatric patients of rectal prolapse (Figure 2). However, the range of 95% CI of several studies included “1” (5, 15, 20). One study revealed the tendency to favor recurrence (13).

## Discussion

In the current meta-analysis, the results showed that the first injection of sclerotherapy might have a higher OR to help the pediatric patients with rectal prolapse achieve a remission status. However, the high heterogeneity and the 95% CI of several studies that included an OR of 1 might bias our findings. In a previously published review (24), they focused on the success rate of a single injection (77%), overall complication rate (14%), and the significant difference between different sclerosing agents (no significant differences). Based on their findings, our meta-analysis enrolled all kinds of sclerosing agents in the first injection of sclerotherapy, which should not have a bias from the different sclerosing agents. Our study also supported that there was a high OR of remission after the first injection of sclerotherapy on the pediatric rectal prolapse. Our results suggested that the first injection of sclerotherapy should play a crucial role in the treatment of pediatric patients with rectal prolapse.

In clinical practice, the management for pediatric rectal prolapse has yet to reach a consensus. There are over 100 surgical procedures in the field of rectal prolapse (25). The majority of infants and children can be spontaneously resolved without an aggressive management. Some patients can resolve after conservative management, such as medical treatment for constipation. However, a subgroup of patients might need aggressive intervention due to persistent symptoms or the lack of spontaneous resolve (2). Therefore, when the clinicians make a clinical judgment, that is, whether they want to take a more aggressive management, sclerotherapy might be an intermediate choice between conservative treatment and surgery.

However, due to the high probability of spontaneous resolution (60–90%) of pediatric rectal prolapse (2), we can observe that it is difficult to enroll a high number of patients for the sclerotherapy, except the studies of Bahador et al. (14) and Malyshev et al. (17). Therefore, the meta-analysis results might be influenced by the two relatively big studies in patient numbers. In addition, the lack of standardization of sclerosing agents, the number, and the location of injections also contributed to the difficulty to standardize the treatment procedures of sclerotherapy. The proportion of patients who would experience a resolution of rectal prolapse with non-operative management remains unknown, even with a recent meta-analysis with such data (24). Another review article showed that the success rate of sclerotherapy in pediatric rectal prolapse was 79.5% (25), which was similar to the meta-analytic data of Hintz et al. (24). However, the review article just used a systematic review strategy to conclude the success rate of sclerotherapy. Therefore, we still need more data to confirm the remission effects of the first injection of sclerotherapy in pediatric rectal prolapse.

There were several limitations in the current study. First, the high heterogeneity and the relatively poor quality of data might bias our findings. Even if the random-effect model can adjust such bias, the influences for our study results were still significant. Second, the lack of demographic data for the “real subgroup” of sclerotherapy in most enrolled studies might limit our ability to analyze the subgroup differences. The lack of demographic data was because we focused on sclerotherapy for pediatric rectal prolapse. However, not all enrolled studies would provide the demographic data “purely” for “sclerotherapy for rectal prolapse,” which is the reason for the lack of some demographic data in Table 1. Third, the lack of a detailed information on subgroups was also an obstacle in performing the subgroup analysis. Fourth, the different kinds of sclerosing agents were included in the current study, which might bias our interpretations. However, the meta-analysis of Hintz et al. suggested that there was no significant difference in the treatment effects between different sclerosing agents (24). Fifth, all the enrolled studies had a retrospective design, which would also influence our meta-analysis results. The references we searched also revealed that the published studies were all retrospective, probably due to the characteristics of the clinical practice of pediatric rectal prolapse, and it might be difficult to enroll such patients in a design of randomized clinical trials or double-blinded trials. Further studies of randomized trials might be warranted to confirm the treatment and remission effects of the first injection of sclerotherapy.

## Conclusion

Despite significant heterogeneity and a relatively low quality of evidence, the first injection of sclerotherapy might demonstrate the therapeutic effects to help the patients of pediatric rectal prolapse achieve remission status. However, more randomized trials with more standardization of sclerotherapy procedures will be warranted in the future to confirm the remission effects of the first injection of sclerotherapy in pediatric rectal prolapse.

## Data Availability Statement

The original contributions presented in the study are included in the article/supplementary material, further inquiries can be directed to the corresponding author.

## Author Contributions

WZ reviewed the abstracts to screen the articles. WZ and YS performed the extraction of clinical outcome data from text, tables, and figures of the enrolled articles independently. MZ checked the manuscript and gave instructions. LL provided the idea of the study design and gave instructions. All authors contributed to the article and approved the submitted version.

## Conflict of Interest

The authors declare that the research was conducted in the absence of any commercial or financial relationships that could be construed as a potential conflict of interest.

## Publisher's Note

All claims expressed in this article are solely those of the authors and do not necessarily represent those of their affiliated organizations, or those of the publisher, the editors and the reviewers. Any product that may be evaluated in this article, or claim that may be made by its manufacturer, is not guaranteed or endorsed by the publisher.
